# Medical genetics and genomic medicine in the United States of America. Part 1: history, demographics, legislation, and burden of disease

**DOI:** 10.1002/mgg3.318

**Published:** 2017-07-16

**Authors:** Carlos R. Ferreira, Debra S. Regier, Donald W. Hadley, P. Suzanne Hart, Maximilian Muenke

**Affiliations:** ^1^ National Human Genome Research Institute National Institutes of Health Bethesda Maryland; ^2^ Rare Disease Institute Children's National Health System Washington District of Columbia

## Abstract

Medical genetics and genomic medicine in the United States of America. Part 1: history, demographics, legislation, and burden of disease.

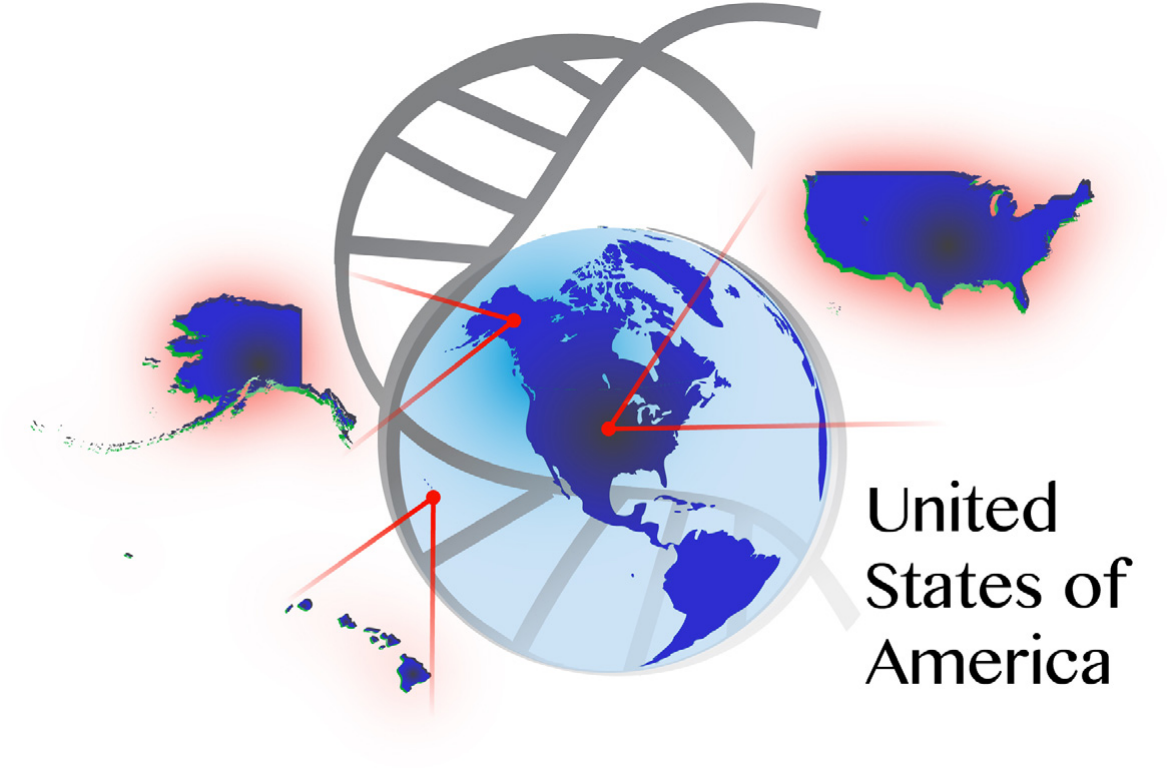

## Introduction

The United States of America became a nation in 1776, when the original 13 colonies declared their independence from the United Kingdom. Over the course of the next several decades, its territory and population continued to expand. Currently, it has a surface area of 9,831,510 square miles (http://data.worldbank.org/; accessed 06/19/2017), with a population of 325,272,738 (https://www.census.gov/; accessed 06/19/2017). Thus, it represents the third country both in area and population. In the 2010 Census, 49.1% of the population was male, and 50.9% of the population was female, while the median age was 37.2 years. Also according to the same census, the Hispanic or Latino ethnicity represented 16.3% of the total population, while non‐Hispanic Whites represented 63.7%. Regarding race, 12.6% of the population was Black or African American, 0.9% was American Indian and Alaska Native, 4.8% was Asian, and 0.2% was Native Hawaiian or other Pacific Islander.

Life expectancy at birth is 78.8 years, more specifically 81.2 years for females and 76.4 years for males (Kochanek et al. 2016). Regarding health care expenditure, the National Health Expenditure Accounts (NHEA) represent the official estimates of health care spending in the country; total nominal US health care spending was $3.2 trillion in 2015, with 17.8% of the gross domestic product devoted to healthcare spending (Martin et al. 2017). Thus, the health care cost is the highest in the world both in totality and per capita.

## History of Genetics in the United States

As with most countries, the history of genetics in the United States is vast. As new perspectives and application of beliefs occurred, what was considered appropriate was then outlawed as barbaric. For example, a dark side of its history is represented by the popularity of the eugenics movement during the early years of the 20th century. In 1907, Indiana became the first state to enact involuntary sterilization legislation—a form of negative eugenics, or improvement of the human race by removing “flawed” individuals from the gene pool (Reilly 2015). Other states soon followed suit, but given their almost compulsory nature, these early laws were legally flawed. As a result of this, Harry H. Laughlin, the Superintendent of the Eugenics Record Office at the Cold Spring Harbor Laboratory, drafted a model eugenic sterilization law that was reviewed by constitutional scholars. In 1924, the Commonwealth of Virginia enacted a law—closely based in Laughlin's model—that allowed eugenic sterilization of people with intellectual disability. This law was put to the test in 1927 with the case of *Buck v. Bell*, when the US Supreme Court upheld a statute permitting compulsory sterilization of the unfit “for the protection and health of the state”. This legal decision opened the floodgates for a barrage of new state legislatures allowing eugenic sterilization. After the eugenics policies of Nazi Germany came to light at the end of World War II, the public perception on eugenics shifted dramatically in the United States, and the number of compulsory sterilizations decreased dramatically.

A more positive aspect of the history of Genetics in this country reveals a multitude of researchers who earned Nobel prizes for their work in the field of Genetics (Table 1), as well as numerous genetic syndromes that were named after American physicians, dentists, or genetic counselors. Table 2 includes a list of eponyms, or persons after whom syndromes have been named.

**Table 1:** Representative American‐born Nobel laureates who worked in the field of genetics

Nobel laureate	Research	Year
Thomas Hunt Morgan	“For his discoveries concerning the role played by the chromosome in heredity”	1933
George W. Beadle	“For their discovery that genes act by regulating definite chemical events”	1958
Edward L. Tatum		
Joshua Lederberg	“For his discoveries concerning genetic recombination and the organization of the genetic material of bacteria”	
Arthur Kornberg	“For their discovery of the mechanisms in the biological synthesis of ribonucleic acid and deoxyribonucleic acid”	1959
James D. Watson	“For their discoveries concerning the molecular structure of nucleic acids and its significance for information transfer in living material”	1962
Marshall W. Nirenberg	“For their interpretation of the genetic code and its function in protein synthesis”	1968
Robert W. Holley		
Alfred D. Hershey	For their discoveries concerning the replication mechanism and the genetic structure of viruses”	1969
David Baltimore	“For their discoveries concerning the interaction between tumour viruses and the genetic material of the cell”	1975
Howard M. Temin		
Daniel Nathans	“For the discovery of restriction enzymes and their application to problems of molecular genetics”	1978
Hamilton O. Smith		
Walter Gilbert	“For their contributions concerning the determination of base sequences in nucleic acids”	1980
Paul Berg	“For his fundamental studies of the biochemistry of nucleic acids, with particular regard to recombinant‐DNA”	
Barbara McClintock	“For her discovery of mobile genetic elements”	1983
Sidney Altman	“For their discovery of catalytic properties of RNA”	1989
Thomas R. Cech		
Philip A. Sharp	“For their discoveries of split genes”	1993
Kary B. Mullis	“For his invention of the polymerase chain reaction (PCR) method”	1993
Eric F. Wieschaus	“For their discoveries concerning the genetic control of early embryonic development”	1995
Edward B. Lewis		
Andrew Z. Fire	“For their discovery of RNA interference ‐ gene silencing by double‐stranded RNA”	2006
Craig C. Mello		
Roger D. Kornberg	“For his studies of the molecular basis of eukaryotic transcription”	2006
Oliver Smithies	“For their discoveries of principles for introducing specific gene modifications in mice by the use of embryonic stem cells”	2007
Carol W. Greider	“For the discovery of how chromosomes are protected by telomeres and the enzyme telomerase”	2009
Thomas A. Steitz	“For studies of the structure and function of the ribosome”	2009
Paul Modrich	“For mechanistic studies of DNA repair”	2015

**Table 2:** Representative American eponyms in the field of genetics

Name	Syndrome	Profession	Reference
George S. Huntington	Huntington disease	General practitioner	Huntington (1872)
William F. Milroy	Milroy disease	Internist	Milroy (1892)
John B. Roberts	Roberts syndrome	Plastic surgeon	Roberts (1919)
Edwin Pyle	Pyle disease	Orthopedic surgeon	Pyle (1931)
Bernard J. Alpers	Alpers progressive infantile poliodystrophy	Neurologist	Alpers (1931)
Myrtelle M. Canavan	Canavan disease	Pathologist	Canavan (1931)
Donovan James McCune	McCune‐Albright syndrome	Pediatrician	McCune (1936); Albright et al. (1937)
Fuller Albright	Albright hereditary osteodystrophy	Endocrinologist	Albright et al. (1942)
Saul W. Jarcho	Jarcho‐Levin syndrome	Internist	Jarcho and Levin (1938)
Paul M. Levin		Neurologist	
Henry H. Turner	Turner syndrome	Endocrinologist	Turner (1938)
Harry F. Klinefelter, Jr	Klinefelter syndrome	Internist	Klinefelter et al. (1942)
Forrest H. Adams	Adams‐Oliver syndrome	Cardiologist	Adams and Oliver (1945)
Clarence P. Oliver		Geneticist	
Kenneth D. Blackfan	Diamond‐Blackfan anemia	Pediatrician	Diamond and Blackfan (1938)
Louis K. Diamond		Hematologist	
Harry Shwachman	Shwachman‐Diamond syndrome	Gastroenterologist	Shwachman et al. (1964)
Conrad M. Riley	Riley‐Day syndrome (familial dysautonomia)	Pediatrician	Riley et al. (1949)
Richard L. Day		Pediatrician	
Loren J. Larsen	Larsen syndrome	Orthopedic surgeon	Larsen et al. (1950)
Eldon J. Gardner	Gardner syndrome	Geneticist	Gardner (1951)
David Bloom	Bloom syndrome	Dermatologist	Bloom (1954)
Sidney Farber	Farber lipogranulomatosis	Pathologist	Farber (1952); Farber et al. (1957)
Thomas P. Kearns	Kearns‐Sayre syndrome	Ophthalmologist	Kearns and Sayre (1958)
George P. Sayre		Ophthalmologist	
Don Marshall	Marshall syndrome	Ophthalmologist	Marshall (1958)
Klaus Patau (German‐born)	Patau syndrome (trisomy 13)	Geneticist	Patau et al. (1960)
George A. Bannayan (Israeli‐born)	Bannayan‐Riley‐Ruvalcaba syndrome	Pathologist	Riley and Smith (1960); Bannayan (1971); Ruvalcaba et al. (1980)
Harris Dr. Riley, Jr.		Pediatrician	
Rogelio H. A. Ruvalcaba (Mexican‐born)		Endocrinologist	
Robert J. Gorlin	Gorlin syndrome	Oral pathologist	Gorlin and Goltz (1960)
Robert W. Goltz	Goltz syndrome	Dermatologist	Goltz et al. (1962)
Sylvester Sanfilippo	Sanfilippo syndrome	Pediatrician	Sanfilippo et al. (1963)
Michael Lesch	Lesch‐Nyhan syndrome	Cardiologist	Lesch and Nyhan (1964)
William L. Nyhan		Geneticist	
Juan Fernandez Sotos	Sotos syndrome	Endocrinologist	Sotos et al. (1964)
Philip M. Marden	Marden‐Walker syndrome	Pediatrician	Marden and Walker (1966)
W. Allan Walker		Gastroenterologist	
Henry T. Lynch	Lynch syndrome	Oncologist	Lynch et al. (1966)
John C. Melnick	Melnick‐Needles syndrome	Radiologist	Melnick and Needles (1966)
Carl F. Needles		Pediatrician	
Leonard O. Langer, Jr	Langer mesomelic dysplasia	Radiologist	Langer (1967)
Angelo M. DiGeorge	DiGeorge syndrome	Endocrinologist	DiGeorge (1968)
Jacqueline A. Noonan	Noonan syndrome	Cardiologist	Noonan (1968)
Frederick Hecht	Hecht syndrome	Geneticist	Hecht and Beals (1969)
Ronald K. Beals	Beals syndrome	Orthorpedic surgeon	Beals and Hecht (1971)
Ann J. Johanson	Johanson‐Blizzard syndrome	Endocrinologist	Johanson and Blizzard (1971)
Robert M. Blizzard		Endocrinologist	
Victor A. McKusick	McKusick‐Kaufman syndrome	Geneticist	Mckusick et al. (1964); McKusick et al. (1968); Kaufman et al. (1972)
Robert L. Kaufman	Kaufman oculocerebrofacial syndrome	Geneticist	Kaufman et al. (1971)
Charles D. Noonan	Saldino‐Noonan syndrome	Radiologist	Saldino and Noonan (1972)
Ronald M. Saldino		Radiologist	
John M. Aase	Aase‐Smith syndrome	Geneticist	Aase and Smith (1968)
Richard E. Marshall	Marshall‐Smith syndrome	Pediatrician	Marshall et al. (1971)
David Weyhe Smith	Mulvihill‐Smith syndrome	Geneticist	Mulvihill and Smith (1975)
John J. Mulvihill		Geneticist	
Carol N. D. Wolcott	Wolcott‐Rallison syndrome	Pediatrician	Wolcott and Rallison (1972)
Marvin L. Rallison		Endocrinologist	
Philip L. Townes	Townes‐Brocks syndrome	Geneticist	Townes and Brocks (1972)
Eric R. Brocks		Ophthalmologist	
William S. Sly	Sly syndrome	Geneticist	Sly et al. (1973)
M. Michael Cohen, Jr	Cohen syndrome	Oral pathologist	Cohen et al. (1973)
Jaime L. Frías (Chilean‐born)	Opitz‐Frias syndrome (Opitz G/BBB)	Geneticist	Opitz et al. (1969)
John M. Opitz (German‐born)	Opitz‐Kaveggia syndrome (FG syndrome)	Geneticist	
Elizabeth G. Kaveggia (Hungarian‐born)		Pediatrician	Opitz and Kaveggia (1974)
David D. Weaver	Weaver syndrome	Geneticist	Weaver et al. (1974)
Robert Neil Schimke	Schimke immunoosseous dysplasia	Geneticist	Schimke et al. (1974)
Ray M. Antley	Antley‐Bixler syndrome	Radiologist	Antley and Bixler (1975)
David Bixler		Oral pathologist	
Victor Escobar	Escobar syndrome	Geneticist	Escobar et al. (1978)
Marvin E. Miller	Miller syndrome	Geneticist	Miller et al. (1979)
Philip D. Pallister	Pallister‐Hall syndrome	Geneticist	Hall et al. (1980)
Judith G. Hall		Geneticist	
Selma A. Myhre	Myhre syndrome	Pediatrician	Myhre et al. (1981)
Helga V. Toriello	Toriello‐Carey syndrome	Geneticist	Toriello and Carey (1988)
John C. Carey		Geneticist	
Robert M. Fineman	Carey‐Fineman‐Ziter syndrome	Geneticist	Carey et al. (1982)
Fred A. Ziter		Neurologist	
Stephen R. Braddock	Braddock‐Carey syndrome	Geneticist	Braddock and Carey (1994)
Ann C. M. Smith	Smith‐Magenis syndrome	Genetic counselor	Smith et al. (1986)
Ruth E. Magenis		Geneticist	
Lisa G. Shaffer	Potocki‐Shaffer syndrome	Geneticist	Potocki and Shaffer (1996)
Lorraine Potocki		Geneticist	
James R. Lupski	Potocki‐Lupski syndrome	Geneticist	Potocki et al. (2000)
Maximilian Muenke (German‐born)	Muenke syndrome	Geneticist	Muenke et al. (1997)
Dorothy K. Grange	Grange syndrome	Geneticist	Grange et al. (1998)

There are also toponymic diseases, that is, conditions named after geographic locations. There are a few examples of genetic terms named after places within the United States. Floating‐Harbor syndrome was named after the Boston Floating Hospital where the condition was first described (Pelletier and Feingold 1973), and the Harbor General Hospital in Torrance, California, where a second patient was reported (Leisti et al. 1975). Tangier disease was named after Tangier Island in Virginia's Chesapeake Bay, where the condition was first identified by NIH researchers (Fredrickson et al. 1961). Ogden syndrome was named after the city of Ogden, Utah, where the original family was first identified (Rope et al. 2011). The Philadelphia chromosome, a short chromosome 22 seen in patients with chronic myelogenous leukemia, was first described by David Hungerford at the Institute for Cancer Research (currently the Fox Chase Cancer Center) and by Peter Nowell at University of Pennsylvania, thus named after the city where both facilities are located (Nowell and Hungerford 1960).

References to genetic conditions can also be found in American artwork. As an example, it has been suggested that the blacksmith depicted in the painting “Among Those Left” by Ivan Albright had Noonan syndrome, based on his low‐set ears, short stature, and pectus deformity. In addition, the great‐grandson of the blacksmith had facial features suggestive of Noonan syndrome, mild pectus deformity, and a dysplastic pulmonary valve leading to pulmonic stenosis (Cole 1980).

Another important aspect in the history of Clinical Genetics in the United States represents the establishments of medical genetics clinics and academic divisions of Medical Genetics, established in 1957 on both coasts of the contiguous United States, by Arno Motulsky at the University of Washington in Seattle, and by Victor McKusick at Johns Hopkins University in Baltimore. Subsequently, Dr. David Weyhe Smith initiated the formal study of dysmorphology in the 1960s, first at the University of Wisconsin in Madison, and later at the University of Washington in Seattle (Frías 2015). In fact, he coined the term dysmorphology (Smith 1966), and in 1970 published the first edition of “Recognizable Patterns of Human Malformation”—widely considered the “bible” of the field. In his honor, a group of his former trainees started the prestigious “David Smith Workshop on Malformations and Morphogenesis”, first held in August 1980, and annually ever since.

## Demographics and Population Diversity

The prevailing hypothesis regarding the population of the Americas proposes that Native American ancestors came from Siberia through the Bering Strait about 14,600 years ago, during the Pleistocene (as during this last glacial period Beringia was not a strait but rather a land bridge). Recent genetic studies have uncovered that this migration from Siberia and East Asia took place about 20,000 years ago—and no earlier than 23,000 years age—and that it diverged into Northern (Athabascan) and Southern (Amerindian) branches around 13,000 years ago (Raghavan et al. 2015). The oldest archeological complex in North America belongs to the Clovis culture, dating from 13,000 to 12,600 years ago. Recently, the whole genome of an infant boy (Anzick‐1) belonging to the Clovis culture was sequenced at an average depth of 14.4×, and it was found that he was closely related to modern Native American populations (Rasmussen et al. 2014). Over the past few centuries, there has been continuous admixture of European, African, and Native American populations in the United States. Recent genetic studies, in fact, show that US populations are more admixed that would be deemed by self‐reported race or ethnicity. Genome‐wide analysis of self‐reported African Americans estimates an average proportion of 73.2% African, 24.0% European, and 0.8% Native American ancestry, while self‐reported Latinos have 18.0% Native American, 65.1% European, and 6.2% African ancestry (Bryc et al. 2015). Approximately 3.5% of self‐reported European Americans have 1% or more African ancestry—representing more than six million Americans who self‐identify as being of European descent, but also have African ancestry. Similarly, as many as five million self‐identified European Americans have at least 1% Native American ancestry (Bryc et al. 2015).

Various genetic isolates exist in the United States. As an example, various genetic disorders have been described more frequently in Inuits from Alaska (Scott 1973), in the Athabaskans from the Southwestern United States such as the Navajo and Apache (Erickson 1999, 2009), and in the Anabaptists, meaning Amish, Mennonites, and Hutterite religious groups that migrated from Europe after the Protestant reformation (Morton et al. 2003; Boycott et al. 2008). A database of genetic disorders prevalent in Anabaptists can be found online at http://www.biochemgenetics.ca/plainpeople/ (Payne et al. 2011).

Regarding consanguinity studies in the general US population, one study based on data collected in 1959 found a rate of 0.08% first‐cousin, 0.02% first‐cousin once‐removed, and 0.11% second‐cousin marriages, for a total of 0.21% consanguinity rate (Freire‐Maia 1968), while a subsequent study found a 0.08% consanguinity rate among Roman Catholics in Wisconsin, USA, in the period from 1976 to 1981 (Lebel 1983). This latter study also showed a progressive decline in rate of first‐cousin marriage over the 20th century: 0.33% from 1902 to 1911, 0.19% from 1912 to1921, and 0.01% from 1972 to 1981. There are, however, specific religious and ethnic minorities with high rates of consanguineous unions. Examples of these include members of the Holiness movement in Kentucky in the 1940s with a consanguinity rate of 18.7% (inbreeding coefficient 0.0061) (Brown 1951), Kansas Mennonites in the 1980s with 33.0% consanguinity (mean inbreeding coefficient 0.0030) (Moore 1987), and Romani Americans in Boston in the 1980s with a 61.9% consanguinity rate (inbreeding coefficient 0.0170) (Thomas et al. 1987).

Second and third cousins may legally marry anywhere in the United States. First cousins can get legally married, without restrictions, in 19 states plus the District of Columbia. In North Carolina, even though first‐cousin marriage is allowed, marriage of double first cousins is specifically prohibited. Twenty‐five states, on the other hand, prohibit first‐cousin marriage altogether. The rest of the six states allow first‐cousin marriage with some restrictions: in Arizona only if at least one partner is infertile, in Illinois only if both partners are over 50 years old or one is infertile, in Maine if they submit to genetic counseling, in Minnesota if permitted by the aboriginal culture of the couple, in Utah if either both parties are at least 65 years old, or if both partners are at least 55 years old and additionally one is infertile, and in Wisconsin if either the female partner is at least 55 years old, or either partner is infertile. In addition, marriage of first cousins once removed is strictly prohibited in four states (Kentucky, Nevada, Ohio and Washington), while it is allowed in Indiana and Wisconsin only if partners are over a certain age or infertile, and is allowed without restrictions in all remaining states (Ottenheimer 1996).

## Burden of Birth Defects and Genetic Diseases in the United States

The number of infant deaths in 2014, when taking into account all causes of infant mortality, was 23,215, for an infant mortality rate of 582.1 infant deaths per 100,000 live births. The leading cause of infant death belonged to the category of congenital malformations, deformations, and chromosomal abnormalities, as it caused 4746 infant deaths, or 20.4% of all infant deaths—for a rate of 119.0 infant deaths per 100,000 live births. Of those, trisomy 21 accounted for 70 infant deaths (rate of 1.8 deaths per 100,000 live births), trisomy18 explained 463 infant deaths (rate of 11.6 deaths per 100,000 live births), while trisomy 13 was the cause of 246 infant deaths (rate of 6.2 deaths per 100,000 live births). Other chromosomal anomalies accounted for 121 infant deaths, for a rate of 3.0 deaths per 100,000 live births (Kochanek et al. 2016). Fortunately, between 1980 and 2001, there was a 46% decline in infant mortality rates secondary to birth defects, primarily related to improved medical care and prevention (Christianson et al. 2006). Racial and ethnic disparities, however, do exist, as postneonatal infant mortality risk is significantly higher in children of non‐Hispanic black mothers for 13/21 birth defects (hazard ratio: 1.3–2.8) and in children of Hispanic mothers for 10/21 defects (hazard ratio: 1.3–1.7) (Wang et al. 2015).

Birth defects are not only the main reason of infant mortality in the United States, but are also associated with significant burden from morbidity. There are 182,786 children born annually with birth defects, for a prevalence of 47.8 per 1000 live births (Christianson et al. 2006). Birth defects and pediatric genetic conditions explain 12% of all pediatric visits to the hospital (CDC, National Center on Birth Defects and Developmental Disabilities). Birth defects accounted for more than 139,000 hospitalizations in 2004, for a hospitalization rate of 47.4 stays per 100,000 persons, and a total of $2.6 billion in hospital costs for that year (Russo and Elixhauser 2007).

Trisomy 21 has an estimated number of 6037 new cases each year (1 in 691 births), with trisomy 18 having an estimated 1109 cases each year (1 in 3762 births), and trisomy 13 with an estimated annual number of 528 new cases, or 1 in 7906 births (CDC, National Center on Birth Defects and Developmental Disabilities). In the case of trisomy 21, the cost per new case has been estimated at $451,000 for 1992, with a total lifetime cost of $1,848,000 (Centers for Disease Control and Prevention (CDC) 1995).

Regarding the cost of birth defects related to preventable conditions, two diagnoses, fetal alcohol syndrome, and neural tube defects have been most characterized. The total US annual cost of fetal alcohol syndrome (FAS) for the year 2002 was estimated at $3.6 billion, while the adjusted lifetime cost for each individual with FAS was $2.0 million (Lupton et al. 2004). An online tool has been devised in order to calculate the prevalence and cost of FAS in local populations (http://www.online-clinic.com/calcs/calc-prev-cost.aspx). Folic acid supplementation of grains led to a one‐third decline in neural tube defects per year, preventing about 500–550 cases of spina bifida each year. Since the societal cost for a child with spina bifida has been estimated at $760,000 per year, while the cost of fortifying the food supply has been calculated at $10 million per year, this is equivalent to an annual $400 million cost savings (Christianson et al. 2006). These two examples show how genetics and public health have worked together to decrease disease and disease costs.

## Genetic Legislation in the United States

The role of the government in protection of genetic information for patients has been a long debated process. The first proposal for a legislation protecting against genetic discrimination in health insurance was the Genetic Information Nondiscrimination Act in Health Insurance of 1995. However, this bill failed to pass either chamber of the 104th Congress. Subsequent iterations of the bill, which eventually expanded to protect against discrimination in employment, were introduced in each subsequent Congress, but none of these bills were passed into law until the 110th Congress, when the bill passed the House with a 414‐1 vote, while it unanimously passed the Senate. After 13 years spent in legislative limbo, the Genetic Information Nondiscrimination Act of 2008 (GINA) was finally signed into law by President George W. Bush on May 21, 2008. Specifically, this legislation prohibits the use of genetic information by health insurers to make decisions about eligibility, coverage, or setting of premiums (Title I), and by employers to make decisions about hiring, salary, promotion, assignment, or firing of employees (Title II). GINA, however, does not provide protection in life, long‐term care, and disability insurance, and does not apply to individuals covered through various forms of military and Federal insurance, such as TRICARE, the Federal Employees Health Benefits (FEHB), and the Veterans Health Administration (VHA).

Although GINA sets the minimum protection to be provided against genetic discrimination, some states have also enacted stricter legislation. To date, 35 states have enacted laws protecting against employment discrimination and 48 states have legislation against insurance discrimination based on genetic information, while 24 states have statutes that limit the use of genetic information by others types of insurance, such as life (17 states), long‐term care (eight states), and disability (17 states) insurance (https://www.genome.gov/27552194/). California passed CalGINA in 2011, which also prohibits discrimination in mortgage lending, housing, education, and other state‐funded programs. The National Human Genome Research Institute (NHGRI) keeps a database of state legislature introduced since 2007 pertaining to genomic information. Known as the Genome Statute and Legislation Database, it is updated on a monthly basis.

Another pertinent legislature includes the Americans with Disabilities Act (ADA), signed into law by President George H. W. Bush on July 26, 1990. This law protects against discrimination based on disability in employment (Title I), public entities (Title II), public accommodations (Title III), and telecommunications (Title IV). Under this law, disability is defined as: (1) a physical or mental impairment that substantially limits one or more major life activities of such individual; (2) a record of such an impairment; or (3) being regarded as having such an impairment. The ADA Amendments Act of 2008 (ADAAA) broadened coverage to protect any individual facing discrimination on the basis of disability. The Equal Employment Opportunity Commission (EEOC) provides a list of conditions that should be regarded as disabilities, including but not limited to deafness, blindness, intellectual disability, missing limbs, autism, or epilepsy. It should be noted, however, that the ADA does not protect from discrimination based on genetic information alone, but exclusively based on impairment.

## Concluding Remarks

By understanding the history of genetics in the United States, we are best able to understand its future. In this two part series, we have begun this discussion and included the demographics, history of genetics, and the economic burden of genetic diseases and birth defects.

The second part of this series will address the current status of prenatal testing, reproductive options and reproductive law in the country, as well as newborn screening, genetic services, rare disease registries, and education and training in the field of genetics.

## Online Resources

CDC—National Center on Birth Defects and Developmental Disabilities. Homepage: https://www.cdc.gov/ncbddd/birthdefects/index.html. June 15, 2017.

## Conflict of Interest

None declared.
